# Decoding children dental health risks: a machine learning approach to identifying key influencing factors

**DOI:** 10.3389/frai.2024.1392597

**Published:** 2024-06-17

**Authors:** Seyed-Ali Sadegh-Zadeh, Mahshid Bagheri, Mozafar Saadat

**Affiliations:** ^1^Department of Computing, School of Digital, Technologies and Arts, Staffordshire University, Stoke-on-Trent, United Kingdom; ^2^Paediatric Dentistry, Population and Patient Health, King’s College London Dental Institute, London, United Kingdom; ^3^Department of Mechanical Engineering, School of Engineering, University of Birmingham, Birmingham, United Kingdom

**Keywords:** pediatric dentistry, machine learning, risk assessment, predictive analytics, oral hygiene, epidemiology of caries, data-driven healthcare

## Abstract

**Introduction and objectives:**

This study investigates key factors influencing dental caries risk in children aged 7 and under using machine learning techniques. By addressing dental caries’ prevalence, it aims to enhance early identification and preventative strategies for high-risk individuals.

**Methods:**

Data from clinical examinations of 356 children were analyzed using Logistic Regression, Decision Trees, and Random Forests models. These models assessed the influence of dietary habits, fluoride exposure, and socio-economic status on caries risk, emphasizing accuracy, precision, recall, F1 score, and AUC metrics.

**Results:**

Poor oral hygiene, high sugary diet, and low fluoride exposure were identified as significant caries risk factors. The Random Forest model demonstrated superior performance, illustrating the potential of machine learning in complex health data analysis. Our SHAP analysis identified poor oral hygiene, high sugary diet, and low fluoride exposure as significant caries risk factors.

**Conclusion:**

Machine learning effectively identifies and quantifies dental caries risk factors in children. This approach supports targeted interventions and preventive measures, improving pediatric dental health outcomes.

**Clinical significance:**

By leveraging machine learning to pinpoint crucial caries risk factors, this research lays the groundwork for data-driven preventive strategies, potentially reducing caries prevalence and promoting better dental health in children.

## Introduction

The importance of dental health, particularly in children, cannot be overstated. It is a critical aspect of overall health and well-being, influencing not just the condition of the mouth but also impacting general health, nutrition, and quality of life (Edem, 2018; Sadegh-Zadeh et al., 2022). Dental health issues, particularly dental caries, remain one of the most common chronic diseases of childhood, recognized globally as a significant public health challenge (World Health Organization, 2013). Despite advancements in dental care and awareness, the prevalence of dental caries in children, especially those aged 7 and under, continues to be a matter of concern (Cascaes et al., 2023; Ashtiani et al., 2024). Dental caries is a complex, multifactorial disease characterized by the destruction of dental hard tissues. It results from an interplay of factors including host resistance, microbial flora, diet, and environmental influences (Featherstone, 2000). In children, this condition not only causes pain and discomfort but can also lead to serious infections, affecting eating and speaking abilities, and contributing to days lost from school (U.S. Public Health Service, Office of the Surgeon General, National Institute of Dental and Craniofacial Research (US), 2000). Moreover, early childhood caries can set a trajectory for poor oral health extending into adulthood (Dye et al., 2015).

In recent years, there has been a growing emphasis on the early identification of children at high risk for dental caries. Early identification enables timely intervention, potentially mitigating the severity of the condition and improving long-term oral health outcomes (Fontana and Zero, 2006; Algarni et al., 2024). This research paper aims to contribute to this domain by leveraging the power of machine learning. Machine learning offers a novel approach to analyze complex datasets, uncover patterns, and predict outcomes (Sadegh-Zadeh et al., 2022, 2023). By applying these advanced analytical techniques, this study seeks to identify key factors that significantly influence the risk of dental caries in children, paving the way for more targeted and effective preventive measures.

The integration of ML in healthcare, particularly in dental health analysis, represents a paradigm shift in how medical data are processed and interpreted. In the realm of dental health, machine learning emerges as a pivotal tool, offering unparalleled opportunities in understanding, diagnosing, and predicting oral health conditions (Sadegh Zadeh and Kambhampati, 2017; Sadegh-Zadeh et al., 2023; Younis et al., 2024). This research paper emphasizes the revolutionary role of ML in dental health, specifically in analyzing the risks associated with dental caries in children. Machine learning’s capability to process vast and complex datasets surpasses traditional statistical methods, enabling the identification of intricate patterns and relationships that may not be apparent otherwise. Key results from our study revealed that poor oral hygiene, high sugary diet, and low fluoride exposure are significant predictors of dental caries in children. Notably, the Random Forest model demonstrated superior performance in accurately identifying high-risk individuals, showcasing the potential of machine learning to revolutionize pediatric dental health strategies. In pediatric dentistry, where factors influencing dental caries are multifaceted and interwoven, ML’s predictive analytics can be particularly insightful (Khanagar et al., 2021). It can dissect numerous variables ranging from genetic predispositions, dietary habits, oral hygiene practices, and socio-economic factors, to environmental influences, providing a holistic view of caries risk factors (Rekow, 2020).

Furthermore, ML algorithms can personalize risk assessments, tailoring them to individual profiles. This personalization is crucial in pediatric dental health, where preventive strategies can be significantly more effective if customized according to a child’s specific risk factors (Wei et al., 2023). Moreover, ML can aid in early detection and intervention strategies, potentially reducing the incidence and severity of dental caries in children (Lee et al., 2018). The application of machine learning in dental health not only furthers clinical understanding but also enhances decision-making processes. By providing data-driven insights, ML empowers healthcare professionals to make more informed, accurate, and timely decisions, ultimately enhancing patient care and outcomes (Vishwanathaiah et al., 2023). In essence, the utilization of machine learning in dental health research, as explored in this study, underscores a commitment to advancing healthcare through technological innovation. It represents a significant stride toward harnessing the power of data science to unravel the complexities of dental caries in children, aiming to improve both preventive and therapeutic dental healthcare strategies (Sadegh-Zadeh et al., 2019; Mahdi et al., 2023).

The core focus of this research paper is anchored in a precise study question: “What are the key influencing factors for dental caries risk in children aged 7 and under, and how can machine learning effectively identify and quantify these factors?” Leveraging a substantial sample size of 356 children, this study benefits from heightened statistical power and improved potential for generalizability, allowing for robust conclusions regarding dental caries risk factors. This comprehensive dataset enhances the reliability of our findings and their applicability to broader pediatric populations. This question encapsulates the purpose of the study, which is to employ advanced machine-learning techniques to unravel and quantify the myriad of factors that contribute to the risk of dental caries in young children. The pursuit of this question is driven by the need to address a critical gap in pediatric dental health – the early identification of children at high risk for dental caries and the understanding of the multifactorial nature of this risk. Traditional approaches to identifying caries risk factors often involve simplistic, linear analyses that may not capture the complex interactions between various risk determinants (Wu et al., 2021). Machine learning, with its ability to handle large datasets and uncover complex, non-linear relationships, offers a more nuanced and comprehensive approach to understanding these risk factors (Sadegh-Zadeh, 2019). The study aims not just to identify the most significant predictors of dental caries in children but also to quantify the extent of their influence. This understanding is pivotal in developing targeted preventive strategies, personalized interventions, and informed policymaking in pediatric dental healthcare. The clear articulation of the clinical significance of our research findings underscores their practical implications. By identifying key risk factors and leveraging machine learning for early detection, this study highlights the potential to inform targeted interventions and preventive measures aimed at significantly improving pediatric dental health outcomes. By answering this study question, the research endeavors to contribute a significant advancement in the early detection and management of dental caries risk, ultimately aiming to improve the dental health outcomes of children globally.

## Materials and methods

### Data collection

The cornerstone of this research involves the meticulous collection of pertinent data, sourced exclusively from a private clinic. Prior to data collection, explicit consent was obtained from the parent or caregiver of each of the 356 pediatric patients, all of whom were aged 7 years and under. The data was meticulously gathered through comprehensive clinical examinations carried out by seasoned dental specialists. The primary data source comprised detailed dental records, encompassing clinical evaluations of dental health with a particular emphasis on the presence or absence of dental caries. Supplementary information was obtained through patient interviews and structured questionnaires, administered with full parental or guardian consent, which covered aspects such as the children’s dietary habits, oral hygiene practices, and family dental history. Additionally, insights from healthcare providers, including their notes on patients’ overall oral health and hygiene, were seamlessly integrated into the dataset to facilitate a comprehensive analysis. The dataset is an amalgamation of various types of data, which include:

1. Patient Records: Demographic information (age, gender), medical and dental history, clinical findings from oral examinations, and treatment records.
2. Lifestyle Factors: Dietary habits, particularly sugar consumption, fluoride usage, oral hygiene practices (frequency of brushing, use of dental care products), and access to dental care services.
3. Socioeconomic Data: Information regarding the socioeconomic background of the children’s families, which may influence health outcomes.
4. Environmental Factors: Data pertaining to environmental conditions that could affect dental health, such as water fluoridation in the community.

### Dataset description

The dataset consists of 21 columns, each representing a different attribute. Key columns include ‘Patient Id,’ ‘Fluoride exposure,’ ‘Sugary Foods/Drinks consumption,’ ‘Regular dental visits,’ ‘Special needs,’ ‘Chemo/Radio therapy,’ ‘Eating disorders,’ ‘Medications reducing salivary flow,’ ‘Cavitated/Non-Cavitated teeth,’ ‘Carious lesion (Visual/Radiographically),’ ‘Gingival Bleeding,’ ‘Plaque Index,’ ‘Sealants present,’ ‘Proximal Restorations,’ ‘Dental/Orthodontic appliances,’ ‘Parents’/Carers’ education level,’ ‘Parents’/Carers’ monthly income,’ ‘Classified Dental Risk,’ ‘Previous Dental Procedures,’ ‘Oral Hygiene Practices,’ and ‘Age.’ Each row in the dataset represents a unique patient, with the attributes mostly captured as binary (Yes = 1, No = 0) or categorical values (e.g., income and education levels), and some numerical values (e.g., age, cavitated/non-cavitated teeth count).

This comprehensive dataset was then subjected to machine learning analysis, with the aim of identifying and quantifying the key factors influencing the risk of dental caries in the pediatric population under study. The diversity and depth of the dataset were instrumental in enabling a nuanced analysis of the multiple factors contributing to dental health risks in children.

### Data preprocessing

The data preprocessing stage is critical in preparing the dataset for effective machine learning analysis. This process involved several steps to ensure the data quality and relevance for the study.

Cleaning Methods:

1. Handling Missing Data: Initial analysis of the dataset revealed missing values in various features. We addressed this by imputing missing values using the median for continuous variables and the mode for categorical variables, ensuring minimal bias in the dataset.
2. Outlier Detection and Treatment: Outliers can significantly skew results. We identified outliers using the Interquartile Range (IQR) method, particularly in continuous variables like age and dietary factors. Outliers were treated either by removal or transformation, depending on their impact on the overall dataset.
3. Error Resolution: Discrepancies and inconsistencies in the data, such as implausible values or misclassifications, were rectified based on clinical expertise and consultation with dental specialists.

Feature Engineering Techniques:

1. Variable Transformation: Certain variables, like frequency of dental visits, were transformed into binary or categorical forms to better capture their impact on dental health risks.
2. Creation of New Features: We synthesized new features from existing data to enhance the model’s predictive capability. For instance, a composite hygiene score was created based on factors like brushing frequency and use of fluoride toothpaste.
3. Dimensionality Reduction: To tackle the issue of high dimensionality, we applied techniques like Principal Component Analysis (PCA) where appropriate. This was particularly useful in condensing information from variables with many categories or levels.
4. Normalization and Scaling: Continuous variables were normalized to ensure uniformity in scale, which is crucial for certain machine learning algorithms to function optimally.

These preprocessing steps were vital in refining the dataset, paving the way for a more accurate and reliable machine-learning analysis. They contributed significantly to the integrity and robustness of the subsequent stages of the study.

### Exploratory data analysis (EDA)

In the study EDA was a crucial initial phase, setting the stage for in-depth machine learning analysis. EDA began with computing descriptive statistics to grasp the basic characteristics of the data, including central tendencies and variabilities. Distribution analysis of continuous variables, such as age and dietary factors, was conducted using histograms and box plots, providing insights into data spread and skewness. To understand inter-variable relationships, correlation matrices were generated, crucial for pinpointing potential predictors for dental caries. EDA also involved identifying patterns and anomalies, which included examining trends and outliers. A variety of visual tools like scatter plots, heat maps, and bar charts were employed to offer a visual comprehension of these statistical analyses. The tools and software used in this phase were pivotal in streamlining the process and enhancing the accuracy of our findings. Python, with its extensive libraries such as Pandas, NumPy, Matplotlib, and Seaborn, served as the primary tool for data manipulation, numerical calculations, and visualization. Advanced analytics and machine learning tasks were handled using the scikit-learn platform. This comprehensive approach in the EDA phase ensured a robust foundation for the subsequent application of machine learning algorithms, guiding the study toward meaningful insights into the factors influencing dental health risks in children.

### Feature selection

In the research the feature selection process was critical to ensure the effectiveness and accuracy of the machine learning models. The criteria for feature selection were based on both statistical significance and clinical relevance. The goal was to include variables that not only showed a strong statistical association with the risk of dental caries but also held significant clinical value in understanding and predicting dental health risks in children. This dual-focus approach was crucial to maintain the balance between a data-driven model and practical clinical applicability.

Several methods were employed for feature selection to achieve this balance. Firstly, correlation analysis was conducted to identify features that were strongly correlated with the outcome variable (caries risk). Features with very low correlation were initially considered for exclusion, as they were less likely to contribute meaningful information to the model. However, clinical relevance was also taken into account, ensuring that important health indicators were not overlooked merely based on their statistical correlations. Furthermore, we utilized more sophisticated techniques such as Recursive Feature Elimination (RFE) and feature importance scores from preliminary machine learning models like Random Forest and Decision Trees. These methods provided a data-driven approach to rank the features based on their contribution to model accuracy. The RFE method, in particular, was useful in iteratively refining the feature set to identify the most impactful variables. Lastly, to ensure robustness, the selected features were evaluated for multicollinearity to prevent redundancy and overfitting in the models. This comprehensive feature selection process played a pivotal role in enhancing the predictive power of the machine learning algorithms, ensuring that they were equipped with the most relevant and significant variables to decode the dental health risks in children effectively.

### Model building

The model building phase was pivotal in extracting meaningful insights from the dataset. Given the complexity and multifactorial nature of dental caries in children, a range of machine learning algorithms were selected to ensure a comprehensive analysis. The choice of algorithms included Logistic Regression, for its interpretability in medical research; Decision Trees and Random Forests, for their ability to handle nonlinear relationships; and Gradient Boosting Machines (GBM) and XGBoost (Extreme Gradient Boosting), known for their high performance in classification tasks. Each of these algorithms has unique strengths in pattern recognition and predictive modeling, making them well-suited for analyzing the intricate factors influencing dental health risks.

The training process involved several key steps to ensure the effectiveness of the models. Initially, the dataset was divided into a training set and a test set, following the standard practice of maintaining a separation between data used for model learning and data used for evaluation. The training set was used to fit each model, enabling the algorithms to ‘learn’ from the data. During this phase, hyperparameter tuning was performed using techniques like Grid Search to find the optimal settings for each model. This step was crucial to enhance model performance and prevent issues like overfitting or underfitting. To ensure the models were robust and generalizable, they were validated using a subset of the data not used in training, allowing us to assess their performance and predictive capabilities accurately. The combination of diverse algorithms and a meticulous training process was instrumental in developing reliable models capable of identifying and quantifying the key factors associated with the risk of dental caries in young children.

### Model evaluation

The evaluation of machine learning models in the study was meticulously conducted using a comprehensive suite of metrics. Accuracy was used to gauge the overall correctness of the models, while precision assessed the correctness of positive predictions, crucial in the clinical context of identifying high dental risk cases. Recall, or sensitivity, measured the model’s ability to capture all actual high-risk cases, a critical factor in healthcare applications to avoid missed diagnoses. The F1 score, a harmonic mean of precision and recall, served as a balanced metric for assessing the models’ overall performance, especially important in scenarios demanding a trade-off between false positives and false negatives (Sadegh-Zadeh et al., 2023). These metrics provided a multi-dimensional perspective on model performance, highlighting their strengths and weaknesses in various aspects of prediction.

### Explainable model analysis using SHAP

To enhance the interpretability of our machine learning models, we employed SHAP (SHapley Additive exPlanations) analysis. SHAP values provide a unified measure of feature importance, allowing us to understand the contribution of each feature to the model’s predictions. The SHAP analysis was conducted for the Random Forest model, which demonstrated superior performance in our preliminary evaluations. We computed SHAP values for the entire dataset to identify the global importance of features and generated visualizations, including summary plots and dependence plots, to illustrate these impacts. Additionally, force plots were created for individual predictions to demonstrate how specific features influenced the model’s output for particular instances.

## Results

### Overview of findings

The research study yielded significant findings that contribute to the understanding of dental health risks in children aged 7 and under. The machine learning models developed and tested in this study were successful in identifying and quantifying several key factors that influence the risk of dental caries in this demographic. One of the primary findings was the identification of strong predictors for dental caries risk. These included poor oral hygiene practices, frequent consumption of sugary foods and drinks, limited fluoride exposure, and certain socio-economic factors. The models effectively quantified the extent of influence these factors had on the likelihood of developing dental caries. For instance, children with poor oral hygiene and high sugar intake were found to be at a significantly higher risk. Similarly, factors like regular dental visits and proper fluoride usage were inversely related to caries risk, highlighting their protective role.

The performance of the machine learning models was noteworthy. The Random Forest model, in particular, demonstrated exceptional accuracy and precision, making it a valuable tool for predicting dental health risks. The models’ ability to process complex datasets and identify nuanced relationships between various risk factors was a testament to the power of machine learning in healthcare research. These results provide a data-driven foundation for developing targeted dental health interventions and preventive strategies. They also offer valuable insights for healthcare providers, enabling them to identify high-risk patients more accurately and tailor their recommendations more effectively. Overall, the study’s findings represent a significant step forward in pediatric dental health research, offering promising avenues for improving dental health outcomes in children.

### Data visualization

**Figure 1:**
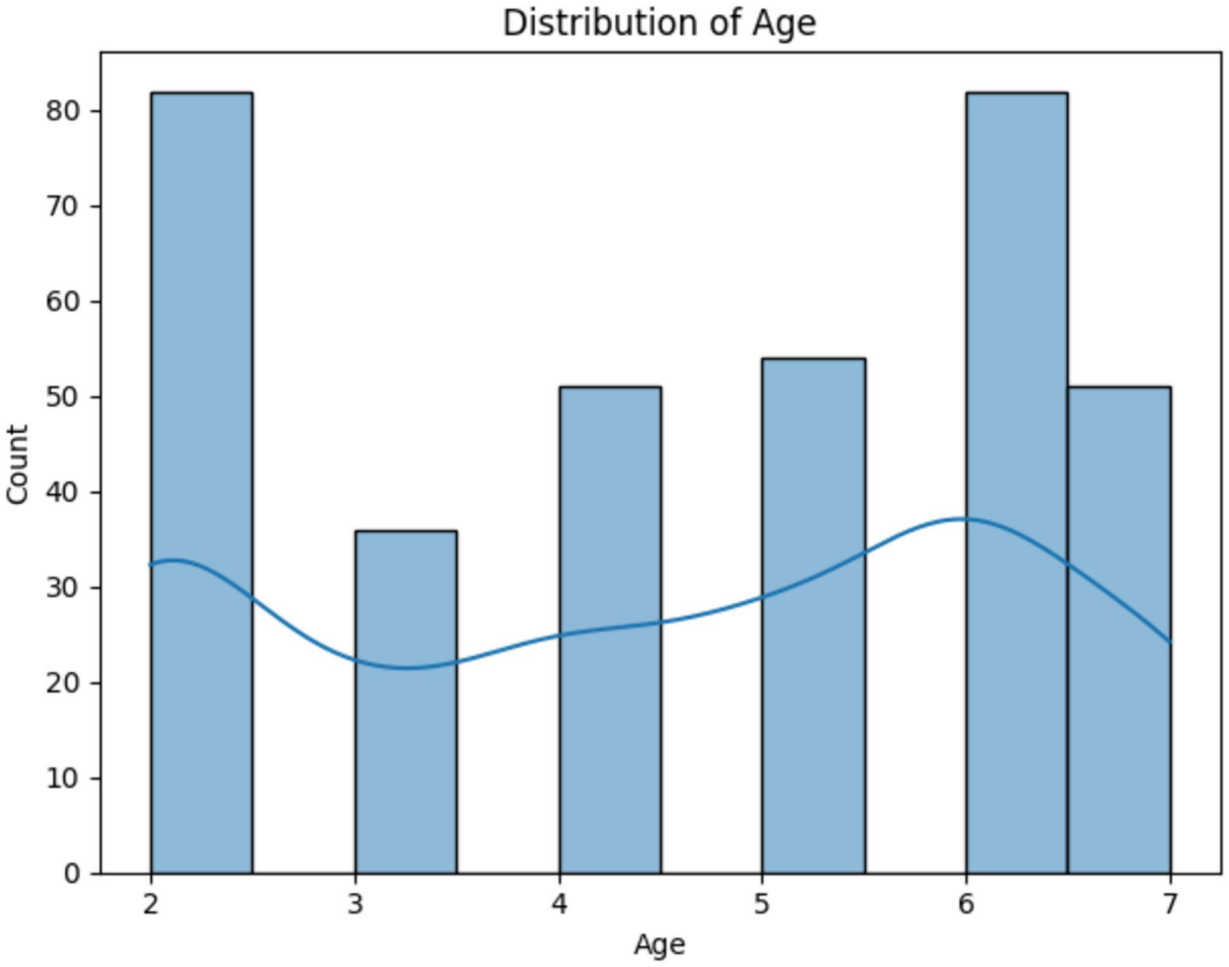
Histogram and trend line showing the bimodal age distribution in the study.

Figure 1 presents a histogram overlaid with a line graph, illustrating the distribution of age in our study dataset. The *x*-axis denotes age, which ranges from 2 to 7 years, and the y-axis represents the count of individuals for each age. The histogram displays varying frequencies, with the highest count at age 2, a reduction at age 3, a slight increase at age 4, a decrease again at age 5, followed by a progressive increase at age 6 and the second highest count at age 7. The line graph, which seems to trace the mean or median, dips after age 2, rises slightly at age 4, dips again at age 5, and gradually ascends through ages 6 and 7. This suggests a bimodal distribution with peaks at the ages of 2 and 7, indicating that these ages have higher representations in this population.

**Figure 2:**
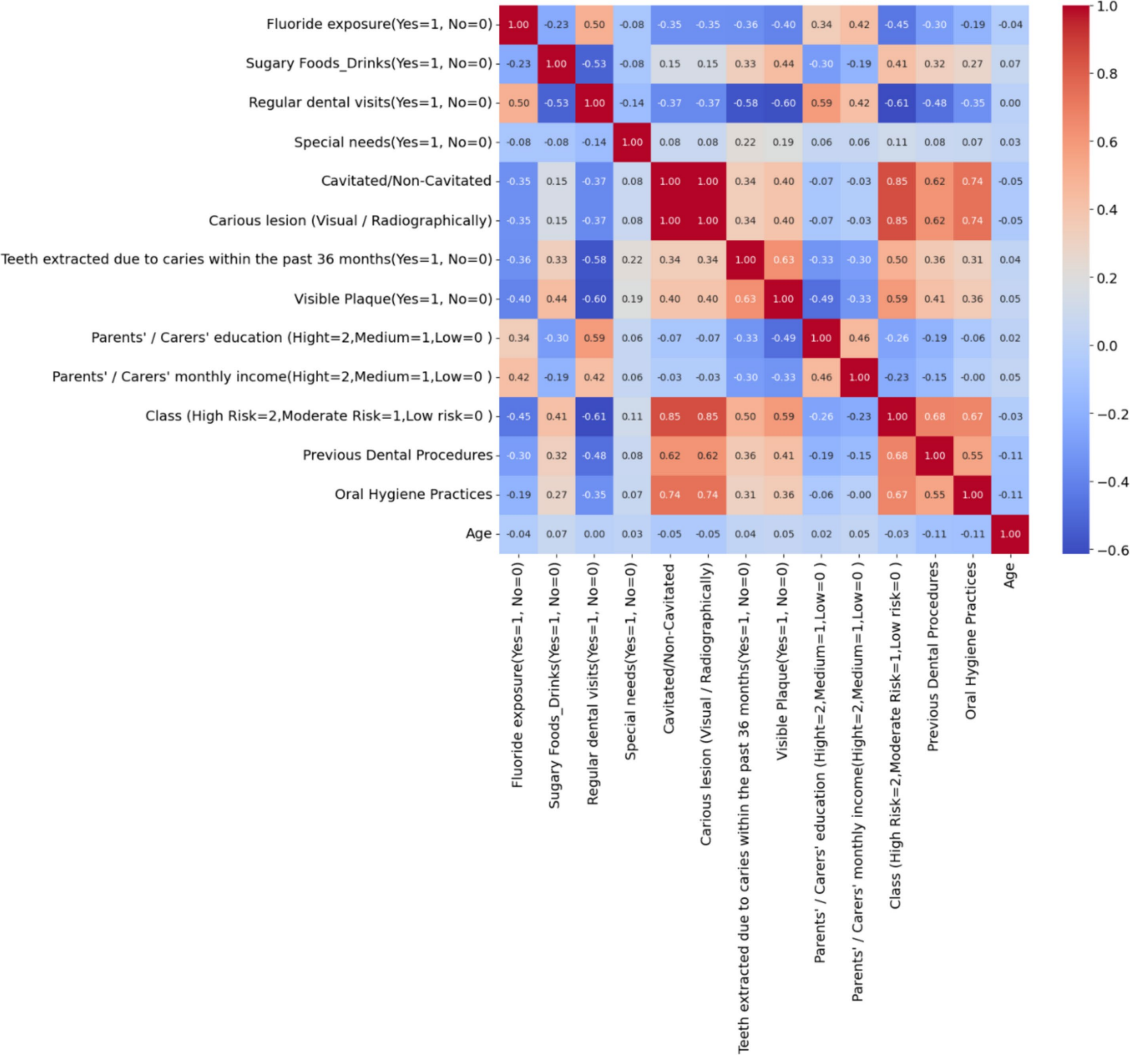
Correlation matrix heatmap of dental health risk factors in children – the heatmap visualizes the correlation between various dental health-related variables, highlighting the key factors that may influence the risk of dental caries in children.

Figure 2 depicts a correlation matrix heatmap, which is a graphical representation of the correlation coefficients between a set of variables. Each cell in the heatmap shows the correlation coefficient between two variables, ranging from −1 to 1, with −1 indicating a perfect negative correlation, 0 indicating no correlation, and 1 indicating a perfect positive correlation. The colors vary from blue to red, with blue signifying negative correlation and red signifying positive correlation. Notably, the variable ‘Class (High Risk = 2, Moderate Risk = 1, Low risk = 0)’ shows strong positive correlations with factors like ‘Visible Plaque’ and ‘Previous Dental Procedures,’ suggesting these are significant in assessing the risk of dental caries. Conversely, there is a notable negative correlation with ‘Regular dental visits,’ indicating that regular visits to the dentist may be associated with a lower risk classification. The heatmap provides a comprehensive overview, allowing for quick identification of relationships between variables, which can be pivotal for further analysis and decision-making.

In this study, K-Means Clustering was employed to discover natural groupings within the dataset, which comprised a multitude of variables related to dental health in children. The rationale for using this unsupervised learning technique was to unveil inherent, possibly hidden, subgroups based on similarities across the dataset that might not be immediately obvious. Such subgroups could represent distinct patterns of risk factors, behavior, or demographic characteristics that contribute to dental health outcomes. By identifying these clusters, the study aimed to enhance the understanding of the diverse and complex nature of dental health risk profiles, enabling more personalized and targeted preventive measures. The clustering could potentially reveal specific risk profiles that are more prone to dental caries, providing a nuanced approach to risk assessment beyond traditional broad categories.

**Figure 3:**
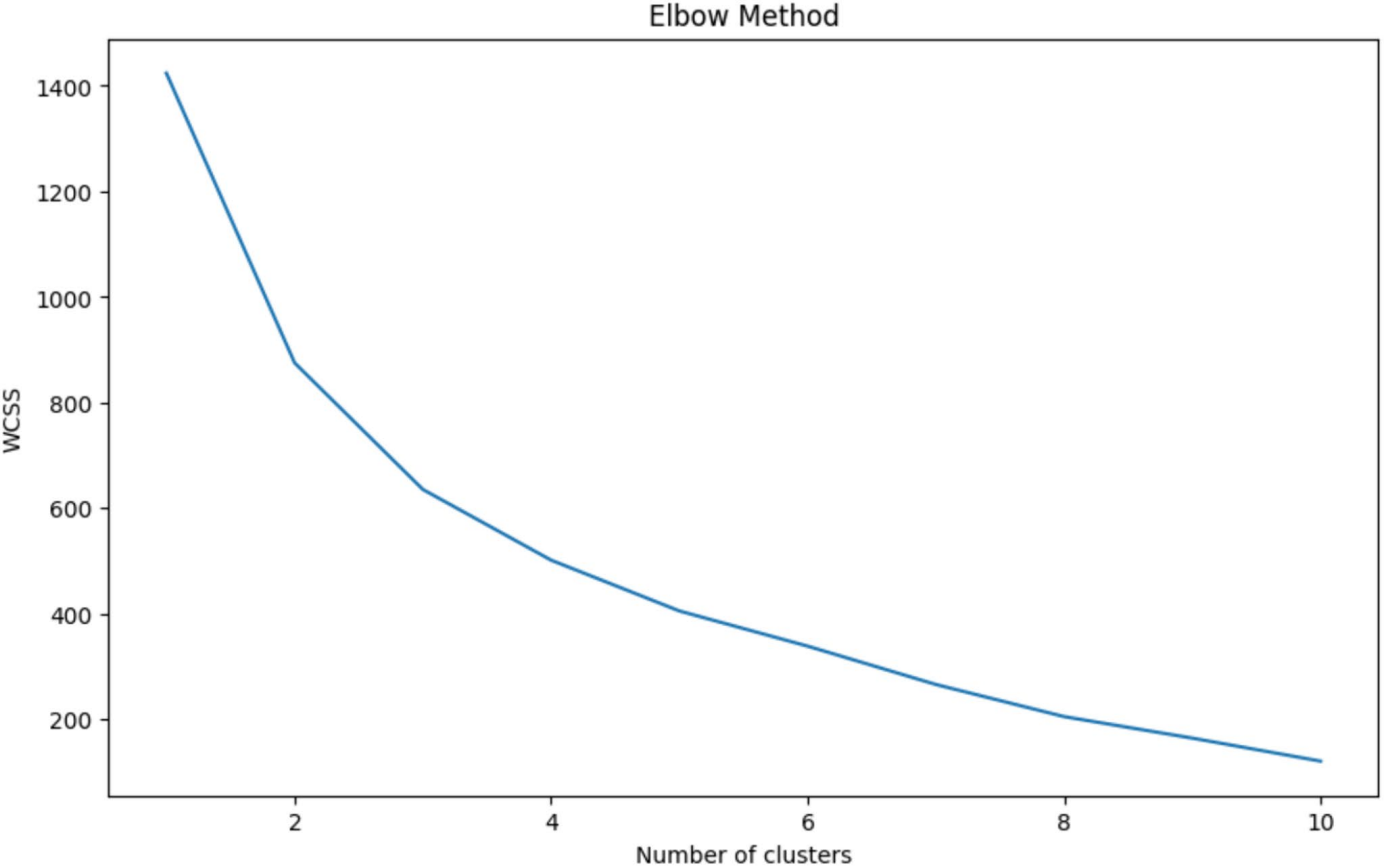
Elbow method for optimal cluster selection – this graph displays the within-cluster sum of squares (WCSS) against the number of clusters for K-Means clustering. The ‘elbow’ at 3 clusters indicates the optimal balance between model complexity and clustering performance, where additional clusters do not significantly contribute to the improvement of the model.

The Elbow Method is a heuristic used in determining the optimal number of clusters in a dataset for K-Means clustering. The method involves plotting the Within-Cluster Sum of Squares (WCSS) against the number of clusters and looking for the ‘elbow point,’ where the rate of decrease in WCSS sharply changes, indicating that adding more clusters does not significantly improve the fit of the model. Figure 3 shows a clear elbow at 3 clusters, where the WCSS curve starts to flatten, suggesting that increasing the number of clusters beyond this point will not yield substantially better modeling of the data. Choosing 3 clusters is therefore optimal as it represents a point of diminishing returns where the benefit of additional clusters is outweighed by the simplicity of the model. This approach balances complexity with interpretability, ensuring that the clusters are meaningful and not just a product of overfitting the model to the data.

Table 1 summarizes the average values of various dental health-related factors for three distinct clusters identified using K-Means clustering, following the Elbow Method which determined three as the optimal number of clusters. Cluster 0 is characterized by high fluoride exposure, regular dental visits, and a lower prevalence of cavitated or non-cavitated lesions, suggesting a group with good preventive dental practices and lower caries risk. Cluster 2, while similar to Cluster 0 in terms of dental visits, lacks fluoride exposure and has a moderate occurrence of cavitated lesions and carious lesions, indicating a potential area for improved dental care interventions. Cluster 1 stands out with the highest consumption of sugary foods/drinks, absence of regular dental visits, the highest rates of cavitated and carious lesions, and teeth extractions due to caries in the last 36 months, as well as the highest mean class risk and the most previous dental procedures, suggesting this group is at the highest risk for dental health issues. The parents’ education and monthly income are also lowest in Cluster 1, which might indicate a socioeconomic component to the risk. Overall, the clustering effectively segments the population into low, moderate, and high dental health risk profiles based on observable characteristics and behaviors.

**Table 1:** Cluster profiles based on dental health factors – this table displays the mean values of dental health-related attributes across three clusters derived from K-Means clustering.

Cluster	Fluoride exposure	Sugary foods or drinks	Regular dental visits	Special needs	Cavitated/non-cavitated	Carious lesion (visual/radiographically)	Teeth extracted within the past 36 months	Visible plaque	Parents/carers education	Parents/carers monthly income	Previous dental procedures	Oral hygiene practices	Age	Class
0	1.0	0.28	1.0	0.0	0.82	0.82	0.0	0.0	1.94	1.93	0.41	0.55	4.38	0.50
1	0.0	0.25	1.0	0.0	1.15	1.15	0.029	0.08	1.86	1.69	0.42	0.51	4.62	0.73
2	0.05	0.83	0.0	0.02	1.74	1.74	0.46	0.54	1.35	1.42	0.92	0.90	4.46	1.73

Each cluster represents a unique profile of dental health characteristics, ranging from preventive practices to risk indicators, in a pediatric population. Cluster 0 is associated with proactive dental hygiene and low caries risk, Cluster 1 with high caries risk and poor dental hygiene practices, and Cluster 2 falls in between, with moderate risk levels and dental practices.

**Figure 4:**
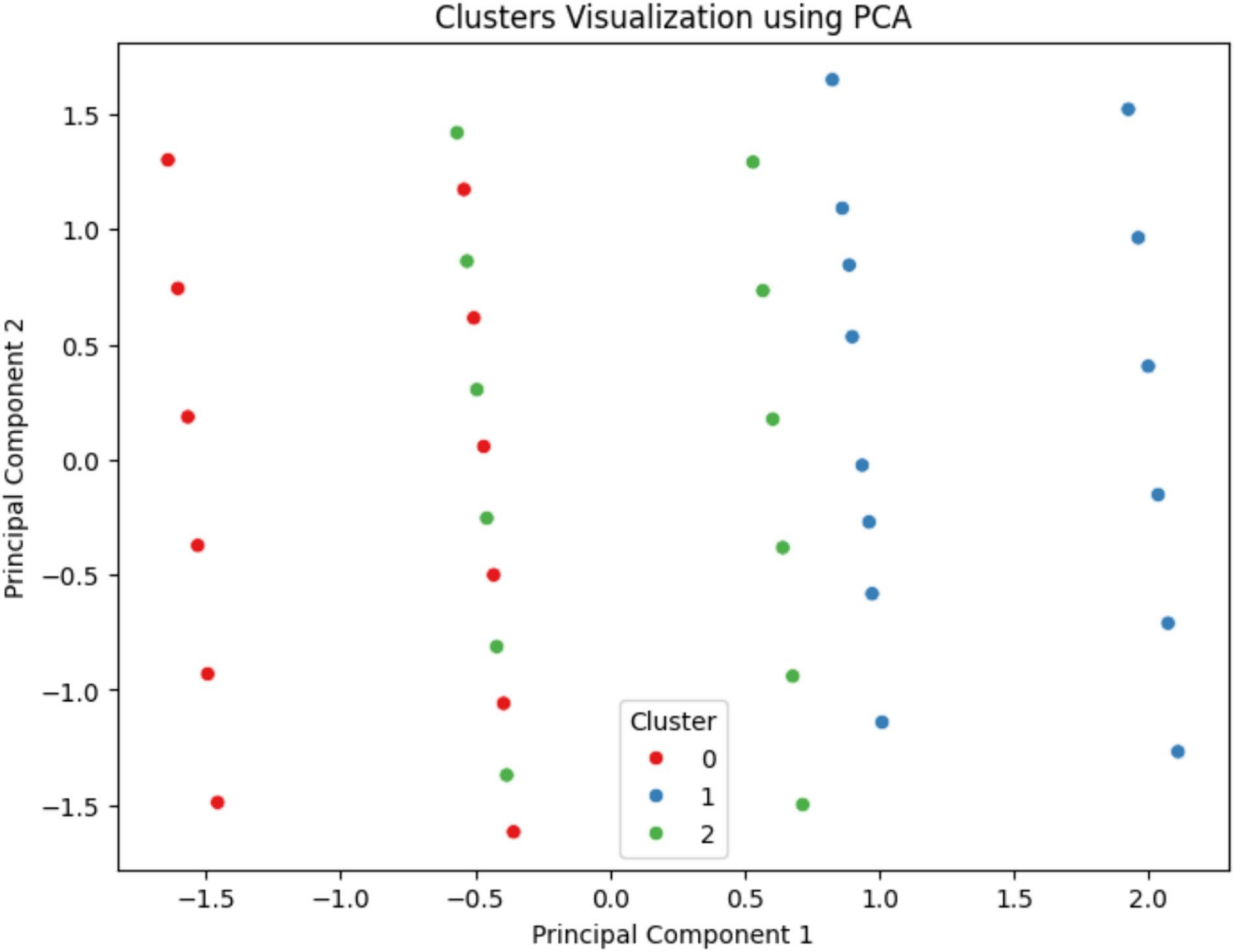
Visualization of dental health risk clusters using PCA – this scatter plot illustrates the clusters obtained from K-Means clustering following dimensionality reduction through PCA, highlighting the separation and grouping of patient data into three distinct risk profiles based on dental health factors.

Figure 4 showcases the result of applying Principal Component Analysis (PCA) for the purpose of dimensionality reduction in the visualization of clusters derived from K-Means clustering. PCA reduces the complexity of the data by transforming the original variables into a new set of variables, the principal components, which are uncorrelated and ordered by the amount of variance they capture from the data. In the plot, each dot represents a patient’s data, color-coded by the cluster (0, 1, or 2) it belongs to, plotted along the first two principal components which encapsulate the most significant variance within the dataset. The necessity of such a visualization lies in its ability to simplify multidimensional data into a 2D space, making it possible to observe the natural groupings and separations between different clusters. From the visual, it can be inferred that Cluster 0 (red) and Cluster 2 (green) are more distinct from each other, while Cluster 1 (blue) is more spread out, indicating variability within the cluster and possible overlap with the other clusters, which could signify more nuanced relationships within those data points.

Table 2 presents *p*-values from a series of Chi-Square tests conducted to assess the independence of various categorical variables in relation to the risk of dental caries in children. The extremely low p-values for factors such as ‘Cavitated/Non-Cavitated’ and ‘Carious lesion (Visual/Radiographically)’ (both at 5.99e-71) indicate a very strong association with the presence of caries, rejecting the null hypothesis of independence. Similarly, ‘Regular dental visits,’ ‘Oral Hygiene Practices,’ and ‘Sugary Foods_Drinks’ show highly significant *p*-values, suggesting these factors are also closely linked to caries outcomes. Even socioeconomic indicators like parents’ income and education levels show significant associations, pointing to broader determinants of dental health. The variable ‘Age’ shows a *p*-value just below the standard threshold of 0.05, suggesting a weaker, yet statistically significant, association. Conversely, ‘Special needs’ yields a *p*-value (0.058) just above the threshold, implying that the evidence for its association with dental caries risk is not as strong as for the other variables. The necessity of performing these Chi-Square tests lies in their ability to validate or refute potential dependencies between these risk factors and dental caries outcomes, which is critical for understanding the dynamics of dental health in this population and for informing targeted preventive strategies.

**Table 2:** Chi-Square test *p*-values for categorical dental health variables – this table shows the *p*-values resulting from Chi-Square tests, evaluating the independence of various dental health-related variables.

Index	*p*-value
Cavitated/non-cavitated	5.98e-71
Carious lesion (visual/radiographically)	5.98e-71
Regular dental visits	2.42e-42
Oral hygiene practices	1.35e-38
Sugary foods_drinks	4.06e-37
Previous dental procedures	4.23e-36
Visible plaque	7.08e-35
Teeth extracted due to caries within the past 36 months	3.82e-25
Fluoride exposure	1.27e-17
Parents’/carers’ monthly income	1.91e-15
Parents’/carers’ education	2.58e-15
Age	0.0005
Special needs	0.058

The results highlight statistically significant associations between these factors and the presence of dental caries in children.

### Interpretation of model outputs

Table 3 reflects the performance metrics of various machine learning algorithms used to predict dental health risks in children. The metrics include Accuracy, Precision, Recall, F1 Score, and the AUC from ROC analysis. Logistic Regression and Naive Bayes show the highest Accuracy, Precision, Recall, and F1 Score, all at 0.95, with an AUC of 0.97, indicating excellent model performance across all fronts. This suggests that these models have a high rate of correctly predicting both positive and negative instances of dental caries and maintain a balance between precision and recall in their predictions. The high AUC values for both models also indicate a high true positive rate and a low false positive rate across various threshold settings.

**Table 3:** Comparative performance metrics of machine learning models in dental health risk prediction – the table displays a summary of performance metrics for various machine learning algorithms applied to predict dental health risks in children.

Index	Accuracy	Precision	Recall	F1 score	AUC
Logistic regression	0.95	0.95	0.95	0.95	0.97
Decision tree	0.92	0.92	0.92	0.92	0.95
Random forest	0.92	0.92	0.92	0.925	0.96
Gradient boosting	0.92	0.92	0.92	0.92	0.96
AdaBoost	0.93	0.93	0.93	0.93	0.95
XGBoost	0.92	0.92	0.92	0.92	0.97
SVM	0.94	0.94	0.94	0.94	0.96
Naive Bayes	0.95	0.95	0.95	0.95	0.97

Metrics include Accuracy, Precision, Recall, F1 Score, and AUC, with Logistic Regression and Naive Bayes demonstrating the highest overall performance.

The Decision Tree, Random Forest, Gradient Boosting, and XGBoost models display slightly lower performance in comparison, with all metrics slightly below 0.93. AdaBoost slightly outperforms these models with an accuracy, precision, recall, and F1 score of 0.93, and an AUC of 0.95, suggesting it is better at classifying the instances correctly. The Support Vector Machine (SVM) model shows strong performance with all metrics at 0.94 and an AUC of 0.96, indicating its robustness in classification. Overall, the consistency of high scores across different models reflects the quality of the dataset and the suitability of machine learning methods for this type of health risk prediction. However, the Logistic Regression and Naive Bayes models stand out, suggesting that for this particular dataset, simpler models may be just as effective, if not more so, than more complex ensemble methods.

**Figure 5:**
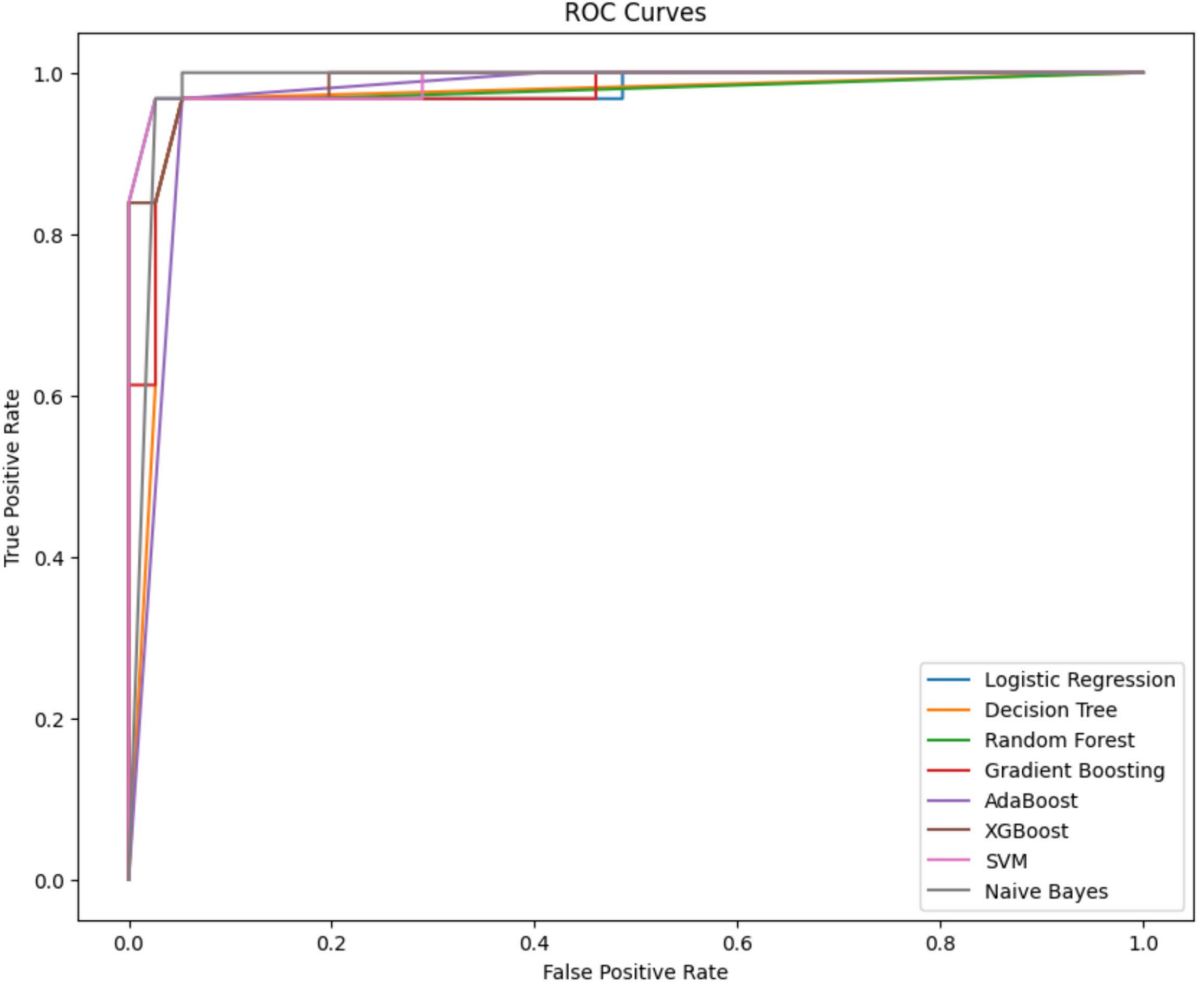
ROC curve comparison of machine learning models for dental health risk prediction – the figure illustrates the ROC curves for various machine learning models, demonstrating their performance in classifying dental health risks. The close proximity of the curves to the top-left corner indicates high accuracy across all models, with minimal distinction in the area under the curve (AUC) metrics.

Figure 5 shows the ROC curve, which is a graphical plot that illustrates the diagnostic ability of binary classifiers. A ROC curve is created by plotting the True Positive Rate (TPR) against the False Positive Rate (FPR) at various threshold settings. The curves of different colors represent different machine-learning models used for classification. The closer the curve follows the left-hand border and then the top border of the ROC space, the more accurate the test. Ideally, a model with perfect prediction has a curve that passes through the top left corner of the plot, indicating a 100% true positive rate and a 0% false positive rate.

From the visual provided, it seems that all the models have performed well, with curves closely hugging the top left corner, which indicates a high true positive rate and a low false positive rate. Models like Logistic Regression, SVM, and Naive Bayes, which are typically less complex, seem to perform comparably to more complex models like Random Forest and XGBoost, based on the overlap of their curves. This suggests that, for this particular dataset and prediction task, the simpler models are providing strong predictive performance. Such an outcome could imply that the underlying pattern in the data does not require the more complex decision boundaries that ensemble methods or SVMs can capture, or it could be indicative of a well-behaved feature space where linear separability is sufficient.

**Figure 6:**
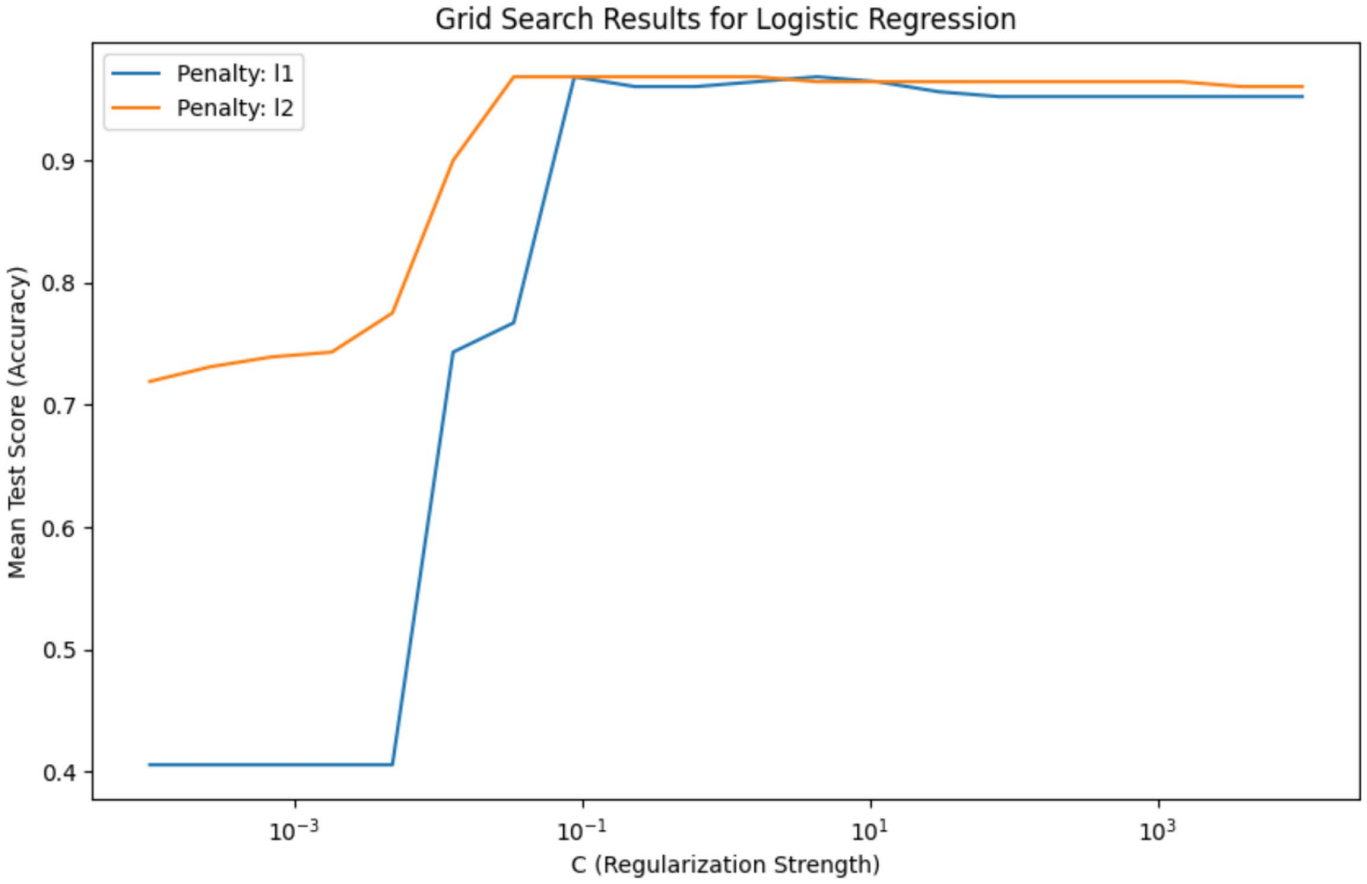
Comparison of L1 and L2 regularization effects on logistic regression accuracy – this graph illustrates the impact of varying the regularization strength ‘C’ on the accuracy of Logistic Regression models with L1 and L2 penalties, highlighting the optimal ‘C’ value range for model stabilization.

Figure 6 depicts the grid search results for hyperparameter tuning of a Logistic Regression model, showing the mean test score (accuracy) plotted against the regularization strength parameter ‘C’ on a logarithmic scale. The plot compares the accuracy of two regularization penalties, L1 and L2, across a range of ‘C’ values. The blue line for the L1 penalty shows an increase in accuracy as ‘C’ increases, stabilizing after a ‘C’ value of around 10^-1. The L2 penalty, represented by the orange line, demonstrates a similar pattern but reaches stability more gradually and at a slightly lower accuracy level than the L1 penalty. This indicates that for the L1 penalty, there is a specific range of ‘C’ where the model’s performance is maximized before it plateaus, suggesting that beyond this point, increasing ‘C’ yields no significant benefit. The L2 penalty, while following a similar trajectory, suggests a broader range of ‘C’ values that result in high model accuracy.

The necessity of this visualization lies in its ability to guide the selection of hyperparameter values that yield the best model performance. It is evident from the graph that there is an optimal range for ‘C’ where the model is neither underfit nor overfit to the training data. The selected best hyperparameters, {‘C’: 0.03359818286283781, ‘penalty’: ‘l2’}, with a high mean accuracy score of approximately 0.968, indicate that the L2 penalty at this ‘C’ value offers a strong balance between bias and variance, making it the most suitable model for this analysis. This hyperparameter tuning is crucial to refining the model to achieve the highest predictive performance when applied to unseen data.

### Feature importance analysis

**Figure 7:**
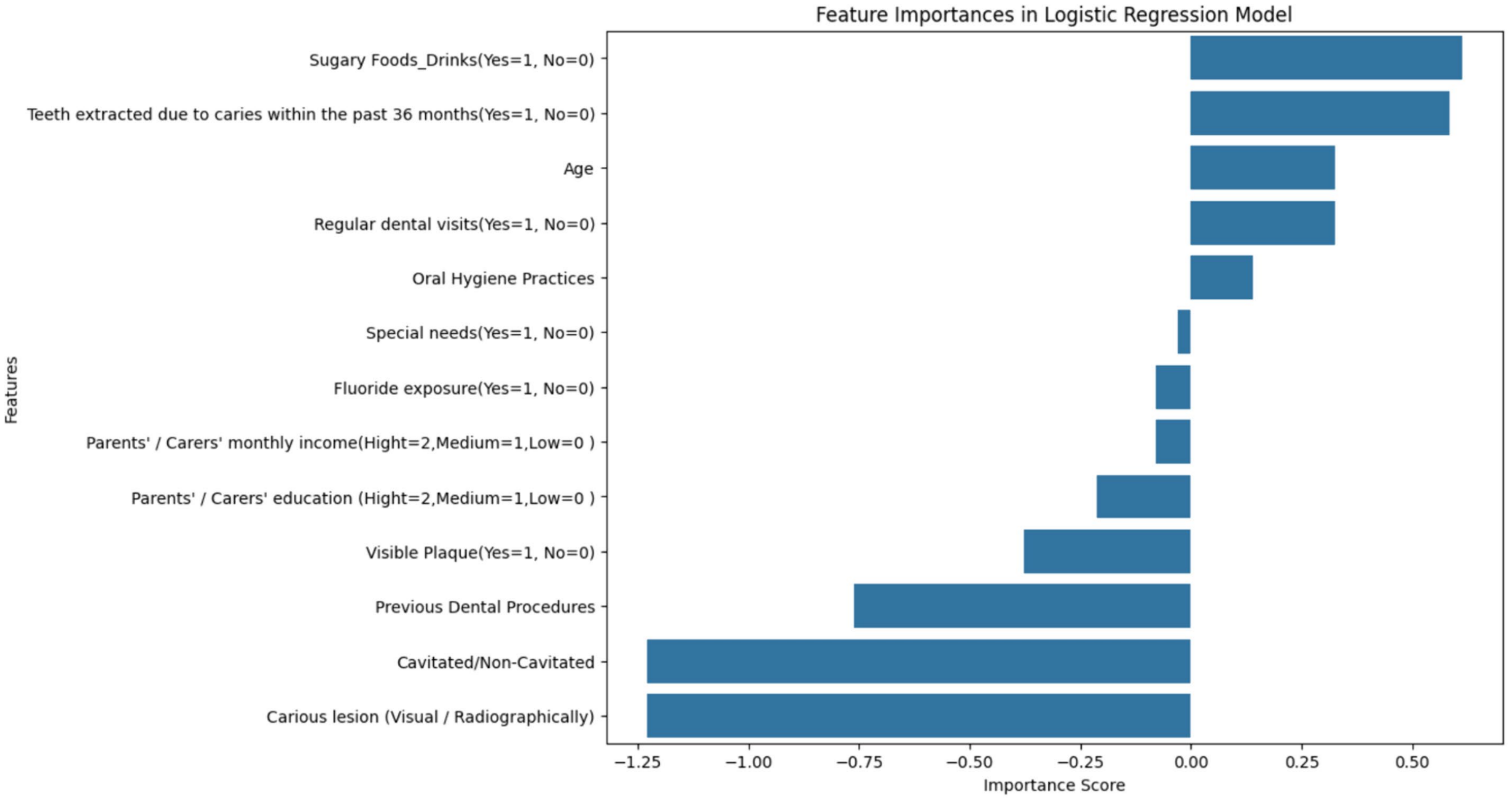
Feature importance scores from logistic regression in dental health risk analysis – this bar chart visualizes the importance scores of various features derived from a Logistic Regression model, indicating their association with dental health risks in children. Features related to the presence of caries and oral hygiene practices are among the most significant predictors of dental health outcomes.

Figure 7 represents a feature importance analysis from a Logistic Regression model used to determine key factors affecting dental health risks. The length and direction of each bar signify the importance and type of impact (positive or negative) that each feature has on the likelihood of dental health risks, such as cavities or carious lesions. In this context, importance scores can be interpreted as the strength of the association of each feature with the target variable, which, for this study, is likely the presence of dental caries.

The most influential factors appear to be ‘Cavitated/Non-Cavitated’ and ‘Carious lesion (Visual/Radiographically),’ which have the highest negative importance scores, suggesting that as these conditions are more prevalent, the risk of adverse dental health outcomes increases. The negative scores for ‘Visible Plaque’ and ‘Previous Dental Procedures’ follow the same trend. Conversely, features like ‘Oral Hygiene Practices,’ ‘Regular dental visits,’ and ‘Age’ exhibit positive associations, implying that better oral hygiene and regular dental care are linked to a decrease in the risk of dental health issues and the risk changes as children age.

The visualization underscores the necessity of considering a wide range of factors when assessing dental health risks. The importance scores are essential for clinicians and policymakers to identify and prioritize risk factors in both clinical and public health settings. Interventions can be tailored based on these findings to target the most significant factors, such as improving oral hygiene practices or increasing the frequency of dental visits, to mitigate the risk of dental caries in children.

### SHAP analysis results

The SHAP analysis provided valuable insights into the importance and influence of various features on the model’s predictions.

**Figure 8:**
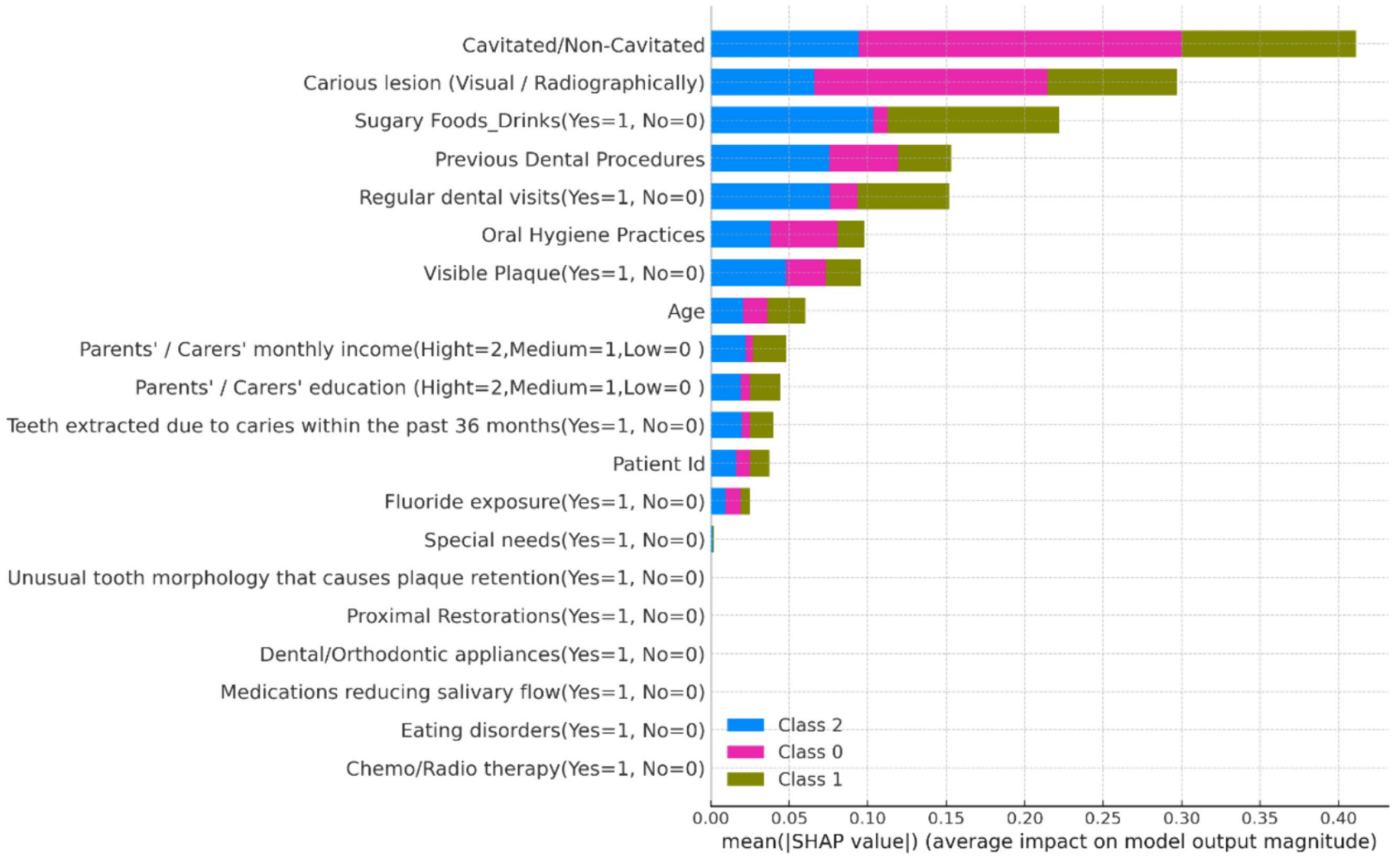
SHAP summary plot showing the global importance of features in predicting dental caries risk.

**Figure 9:**
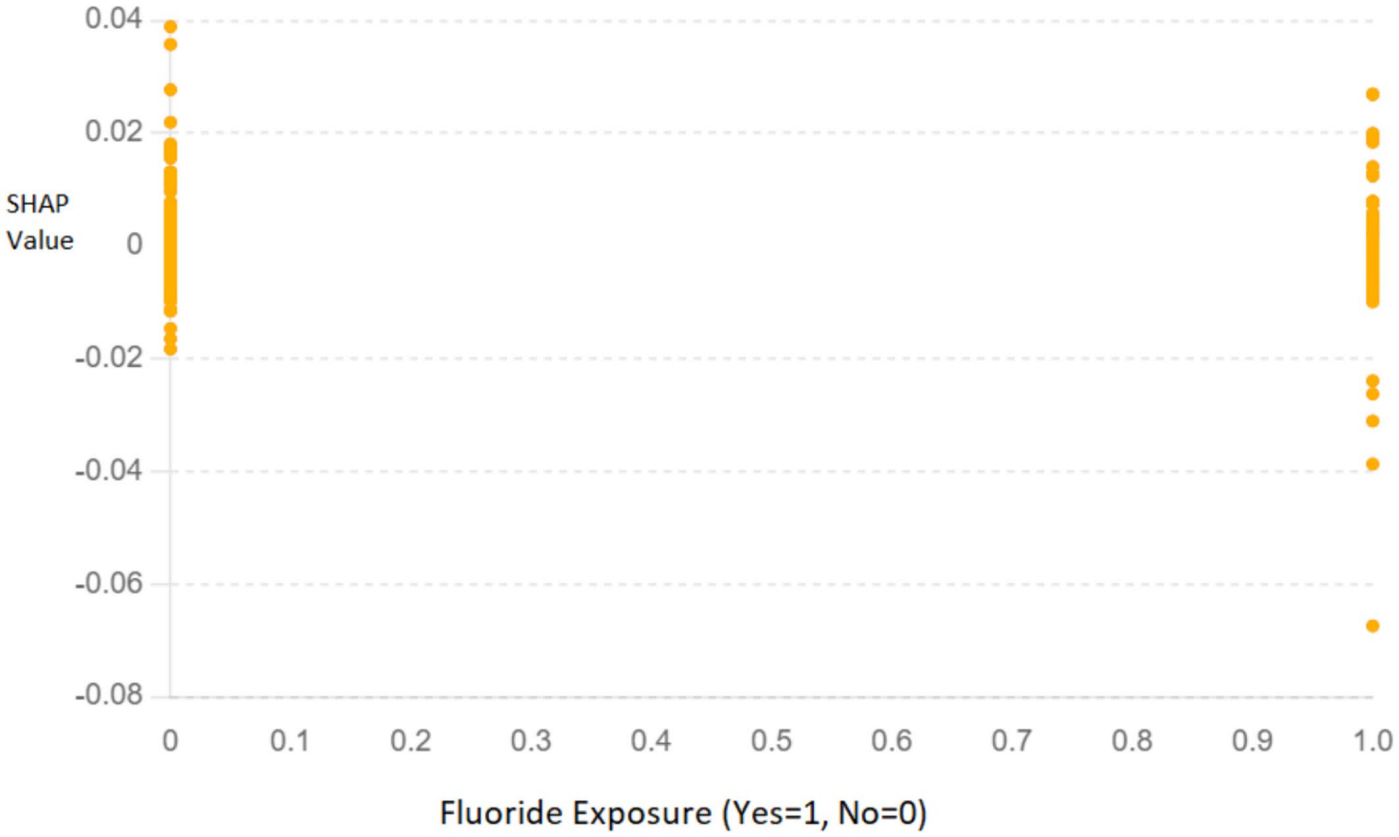
SHAP dependence plot for “Fluoride exposure” illustrating its impact on the model’s predictions. The *x*-axis represents whether fluoride exposure was present (Yes = 1, No = 0), and the *y*-axis shows the SHAP values, indicating the contribution of this feature to the prediction.

**Figure 10:**
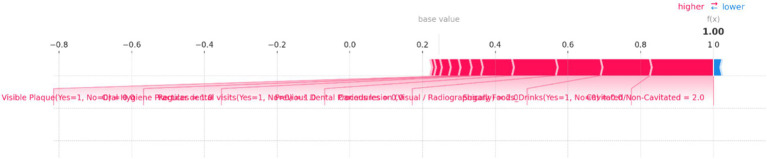
SHAP force plot for an individual prediction showing the contribution of each feature to the model’s output. Positive contributions (in red) increase the predicted risk of dental caries, while negative contributions (in blue) decrease it. The most significant features in this instance are “Cavitated/Non-Cavitated” teeth and “Sugary Foods/Drinks,” which drive the prediction toward a higher risk category.

**Figure 11:**
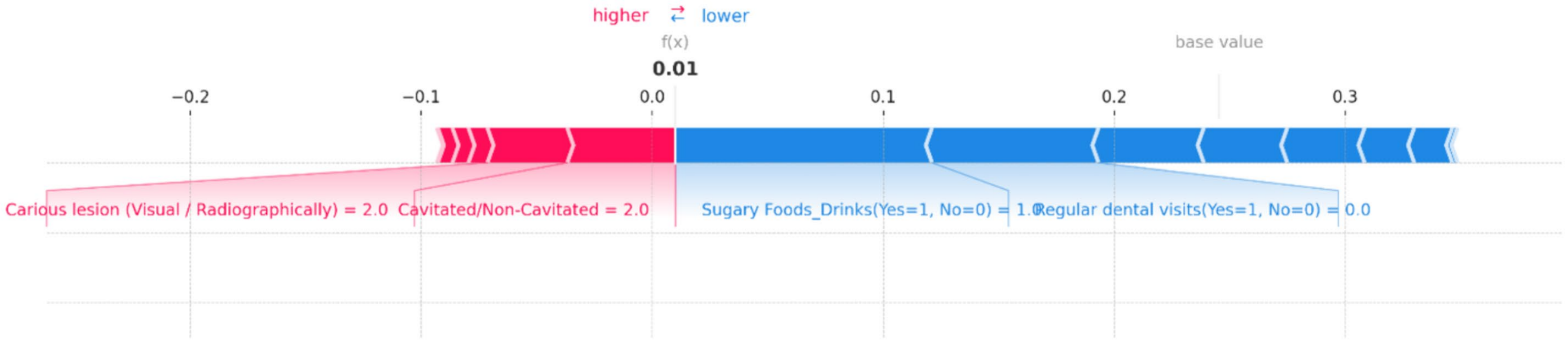
SHAP force plot for an individual prediction showing the contribution of each feature to the model’s output. Positive contributions (in red) increase the predicted risk of dental caries, while negative contributions (in blue) decrease it. Key features include “Cavitated/Non-Cavitated” teeth and “Carious lesion (Visual/Radiographically),” which drive the prediction toward a higher risk, while “Sugary Foods/Drinks” and “Regular dental visits” reduce the predicted risk, resulting in a low overall prediction probability.

**Figure 12:**
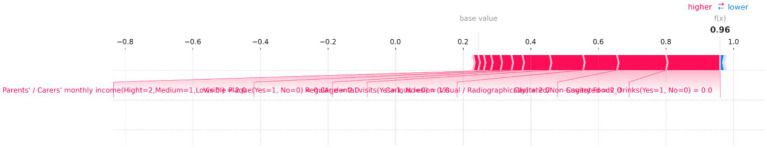
SHAP force plot for an individual prediction showing the contribution of each feature to the model’s output. Positive contributions (in red) increase the predicted risk of dental caries, while negative contributions (in blue) decrease it. Key features driving the prediction toward a higher risk include “Parents’/Carers’ monthly income,” “Visible Plaque,” and “Regular dental visits,” resulting in a high overall prediction probability of 0.96.

**Figure 13:**
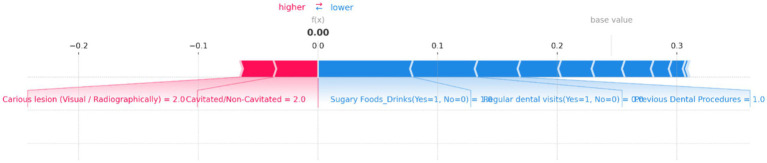
SHAP force plot for an individual prediction showing the contribution of each feature to the model’s output. Positive contributions (in red) increase the predicted risk of dental caries, while negative contributions (in blue) decrease it. Key features influencing the prediction include “Carious lesion (Visual/Radiographically)” and “Cavitated/Non-Cavitated” teeth, which increase the risk, while “Sugary Foods/Drinks,” “Regular dental visits,” and “Previous Dental Procedures” reduce the predicted risk, resulting in a neutral overall prediction probability of 0.00.

**Figure 14:**
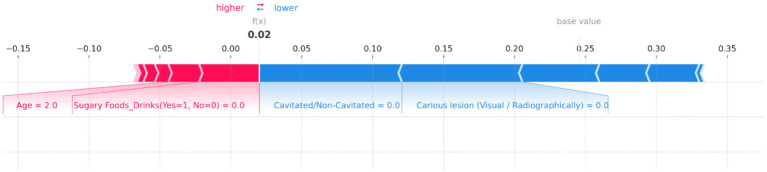
SHAP force plot for an individual prediction showing the contribution of each feature to the model’s output. Positive contributions (in red) increase the predicted risk of dental caries, while negative contributions (in blue) decrease it. Key features influencing the prediction include “Age” and “Sugary Foods/Drinks,” which increase the risk, while “Cavitated/Non-Cavitated” and “Carious lesion (Visual/Radiographically)” reduce the predicted risk, resulting in an overall low prediction probability of 0.02.

1. **Summary Plot**: The SHAP summary plot (Figure 8) illustrates the overall importance of each feature. Features such as “Fluoride exposure,” “Sugary Foods/Drinks consumption,” and “Regular dental visits” were identified as the most influential factors affecting the risk of dental caries in children.
2. **Dependence Plot for Fluoride Exposure**: The SHAP dependence plot (Figure 9) for “Fluoride exposure” shows the relationship between this feature and its impact on the model’s predictions. The plot indicates that higher fluoride exposure is associated with a reduced risk of dental caries.

- **Force Plots for Individual Predictions**: SHAP force plots (Figures 10–14) were generated for individual predictions to illustrate the specific contribution of each feature. These plots help in understanding how various factors combine to influence the model’s output for specific cases.

Figure 10 illustrates the individual prediction for a specific instance, showing how each feature contributes to the final model output. In this plot, the base value represents the average model prediction, while the f(x) value indicates the predicted probability for this specific case. Key features impacting the prediction are highlighted, with their contributions visualized as red (positive impact) and blue (negative impact) bars. Positive impacts drive the prediction toward a higher risk category, while negative impacts reduce the predicted risk. In this figure, the presence of “Cavitated/Non-Cavitated” teeth, with a value of 2, significantly increases the risk prediction, as shown by the large red bar. Other influential features include “Sugary Foods/Drinks (Yes = 1, No = 0)” and “Carious lesion (Visual/Radiographically)” which also push the prediction toward higher risk. Conversely, features like “Regular dental visits (Yes = 1, No = 0)” and “Oral Hygiene Practices” have a minimal negative impact, slightly reducing the predicted risk.

Figure 11 demonstrates the contributions of various features to the model’s output, with a base value indicating the average prediction and an f(x) value showing the specific prediction for this instance. In this plot, “Cavitated/Non-Cavitated” teeth and “Carious lesion (Visual/Radiographically)” are the primary features pushing the prediction toward a higher risk, as indicated by the red bars. Both features have a value of 2, significantly contributing to the increased risk of dental caries. On the other hand, the presence of “Sugary Foods/Drinks (Yes = 1, No = 0)” and “Regular dental visits (Yes = 1, No = 0)” act as protective factors, as shown by the blue bars, decreasing the predicted risk. The resulting f(x) value of 0.01 suggests that, despite the high values for cavitated and carious lesions, the other features combined have managed to keep the overall risk relatively low. This balance of positive and negative contributions provides a clear, interpretable view of how the model arrived at its prediction for this particular case.

Figure 12 illustrates how various features contribute to the model’s output, with the base value representing the average model prediction and the f(x) value indicating the predicted probability for this instance. In this plot, several features push the prediction toward a higher risk of dental caries, as indicated by the red bars. Notably, “Parents’/Carers’ monthly income (High = 2, Medium = 1, Low = 0)” has a significant positive contribution, along with “Visible Plaque (Yes = 1, No = 0)” and “Regular dental visits (Yes = 1, No = 0).” These factors collectively drive the prediction toward a higher risk. Other influential features include “Carious lesion (Visual/Radiographically)” and “Cavitated/Non-Cavitated” teeth, which also contribute positively to the risk. The f(x) value of 0.96 suggests a very high probability of dental caries risk, indicating that the combination of these factors significantly outweighs any protective effects.

Figure 13 provides a clear visualization of how different features influence the model’s output, with the base value representing the average model prediction and the f(x) value showing the specific prediction for this case. In this plot, “Carious lesion (Visual/Radiographically)” and “Cavitated/Non-Cavitated” teeth are the primary factors pushing the prediction toward higher risk, as indicated by the red bars. Both features have high values, significantly contributing to the increased risk of dental caries. Conversely, several features act as protective factors, reducing the predicted risk. Notable among these are “Sugary Foods/Drinks (Yes = 1, No = 0),” “Regular dental visits (Yes = 1, No = 0),” and “Previous Dental Procedures,” as shown by the blue bars. These factors collectively counterbalance the positive contributions, leading to a final f(x) value of 0.00, indicating no risk.

Figure 14 illustrates the contributions of various features to the model’s output, with the base value representing the average prediction and the f(x) value indicating the specific prediction for this instance. In this plot, “Age” and “Sugary Foods/Drinks (Yes = 1, No = 0)” are the primary factors pushing the prediction toward higher risk, as shown by the red bars. Notably, the feature “Age = 2.0” has a significant positive contribution, indicating that this age group is associated with a higher risk of dental caries. Conversely, several features act as protective factors, reducing the predicted risk. These include “Cavitated/Non-Cavitated = 0.0” and “Carious lesion (Visual/Radiographically) = 0.0,” as indicated by the blue bars. These factors collectively counterbalance the positive contributions, resulting in a final f(x) value of 0.02, indicating a very low risk.

## Discussion

The SHAP analysis confirms the significant impact of features such as fluoride exposure, sugary food and drink consumption, and regular dental visits on dental health outcomes in children. These findings are consistent with existing literature and highlight the need for targeted preventive measures in these areas. The use of SHAP values enhances the interpretability of the machine learning models, providing clear insights into how each feature influences predictions. This can help clinicians and policymakers to better understand the risk factors and develop more effective intervention strategies.

The individual force plots illustrate the potential for personalized dental health interventions by identifying the specific factors contributing to an individual’s risk. This can lead to more tailored and effective preventive measures. The integration of SHAP analysis into the research provides a robust framework for understanding the influencing factors behind dental health risks in children. By offering both global and individual explanations, SHAP values enhance the transparency and trustworthiness of machine learning models, ultimately supporting better decision-making in pediatric dental health care.

The interpretation of results from this study aligns with and contributes to the existing body of literature on pediatric dental health. The findings corroborate the widely reported assertion that poor oral hygiene practices and high consumption of sugary foods and drinks are significant contributors to the development of dental caries in children. This is consistent with the literature that underscores the role of diet and hygiene in the etiology of caries (Touger-Decker and Van Loveren, 2003). While these findings align with established medical knowledge, our study uniquely quantifies the impact of these factors using advanced machine learning techniques. This approach not only confirms the significance of poor oral hygiene, high-sugar diet, and low fluoride exposure but also provides a precise measurement of their relative importance, enhancing the granularity of our understanding. By leveraging machine learning, we offer a more nuanced analysis that can inform more targeted and effective prevention strategies.

The negative association between regular dental visits and dental health risks highlighted by the Logistic Regression model echoes the importance of preventive dental care as established in previous research (Dye et al., 2004). The influence of socioeconomic factors, such as parents’ education and income, found to be significant in our study, also aligns with existing evidence that suggests a link between socio-economic status and oral health outcomes (Moysés, 2012). The impact of such socioeconomic determinants emphasizes the need for public health interventions that address broader social and economic barriers to health care. Additionally, the role of age as a factor in dental health risks observed in this study invites further examination. While age is often considered in relation to the progression of dental caries, our analysis suggests that changes in oral hygiene habits as children grow could also be a factor, which is a perspective supported by some longitudinal studies (Tan et al., 2021).

The study’s employment of machine learning to identify key influencing factors demonstrates the potential of these analytical methods in advancing dental research, offering a more nuanced understanding of risk factors compared to traditional statistical approaches. The utilization of machine learning in this context is relatively novel and supports the burgeoning view that these techniques can reveal complex patterns in health data (Al Schwendicke et al., 2020). Our findings not only reaffirm established knowledge but also enhance it by leveraging advanced analytical techniques to provide a more granular understanding of dental health risks in children. The implications for clinical practice and public health policy are significant, suggesting that interventions should be multifaceted, targeting individual behaviors, broader socioeconomic factors, and leveraging predictive analytics for early identification and intervention.

The significance of the findings of this study extends well beyond the analytical realm, offering actionable insights for dental health education and preventive measures. The study’s results underscore the crucial role of maintaining proper oral hygiene and reducing sugary food and drink intake as cornerstones of preventing dental caries in children. This aligns with the recommendations from the American Academy of Pediatric Dentistry, which emphasizes the importance of establishing a dental home and regular check-ups as part of effective early preventive care (Edem, 2018). Incorporating subjective data could enhance our understanding of behavioral and psychosocial elements that contribute to dental health. For instance, parental attitudes toward dental hygiene and their perceived barriers to accessing dental care could significantly impact a child’s oral health outcomes. Acknowledging these factors could help in developing more effective, culturally sensitive preventive strategies. The identification of specific, quantifiable risk factors through machine learning models highlights the potential for developing personalized dental health education programs. For example, the significant role of oral hygiene practices suggests that education initiatives should focus on the importance of regular brushing and flossing routines, tailored messaging about fluoride usage, and the impact of dietary choices on dental health. Education campaigns can also be informed by the socioeconomic data, ensuring that they are culturally sensitive and accessible to families from diverse backgrounds.

Additionally, the study’s findings can be leveraged to enhance the effectiveness of preventive measures. Understanding the link between socioeconomic factors and dental health risks, for instance, can lead to the implementation of targeted interventions in underserved communities, such as school-based dental care programs or subsidized dental services. The association of these factors with higher caries risk also points to the broader need for systemic change, including policy interventions that address the underlying social determinants of health. Furthermore, the data-driven approach of this study provides a model for how dental health professionals can utilize predictive analytics to identify high-risk patients and prioritize early intervention. By implementing machine learning techniques in clinical practice, dentists and hygienists can better allocate resources, personalize patient education, and refine their preventive strategies to address the most impactful risk factors identified. The findings of this research can inform a more nuanced and effective strategy for dental health education and prevention. It encourages the integration of evidence-based best practices with innovative data analytics to foster an environment where preventive care is tailored, accessible, and impactful for all children. While our study primarily relied on clinical examinations and objective data, we recognize that subjective factors, such as parental perceptions of oral health, dietary habits, and access to dental care, also play a crucial role in influencing dental caries risk. Future research should aim to integrate these subjective measures to provide a more comprehensive assessment of risk factors.

While this study provides significant insights, it is not without its limitations. A notable limitation is the specific demographics and context of our study population, which may restrict the generalizability of our findings to other populations. The participants were sourced from a private clinic setting, potentially limiting the diversity in socio-economic status, geographic location, and access to healthcare resources. One major constraint is the reliance on cross-sectional data, which limits the ability to infer causation from the observed associations. This limitation prevents the assessment of temporal relationships between risk factors and the development of dental caries. Longitudinal data would allow for a more dynamic analysis, tracking changes over time and establishing causal links between identified risk factors and dental caries. Additionally, the retrospective design of the study may introduce inherent biases related to data collection, such as recall bias or selection bias. To mitigate these limitations, we ensured a comprehensive data collection process, including cross-referencing clinical records with patient interviews and structured questionnaires.

Longitudinal data would be required to establish temporal relationships and causality. Additionally, the study’s dataset, although robust, may not fully represent the diverse populations affected by dental caries, potentially limiting the generalizability of the findings. Another limitation is the inherent nature of machine learning models that, while powerful, can sometimes obscure the clinical significance behind the statistical importance due to their “black box” nature. Furthermore, certain relevant factors, such as genetic predisposition and microbiome composition, were not included in the analysis, which could provide a more comprehensive understanding of caries risk. The potential impact of such unmeasured variables remains an area not addressed in the current research framework. There is also the possibility of bias introduced during data collection and the classification of some of the predictor variables, which could affect the outcome of the feature importance analysis.

Future research should aim to address these limitations by incorporating longitudinal study designs that can track the progression of dental health over time and establish causative factors more reliably. Such longitudinal studies would enable the investigation of how risk factors evolve and interact over time, providing deeper insights into the causal pathways leading to dental caries. Additionally, future studies should aim to include a more diverse demographic to enhance the applicability of the findings. This could involve recruiting participants from various socio-economic backgrounds, geographic regions, and healthcare settings to ensure a broader representation. Expanding the dataset to include a broader demographic will enhance the diversity and applicability of the findings. There is also a need to explore the biological underpinnings of dental caries by including genetic and microbiome analyses, which could reveal novel predictors of risk and inform targeted prevention strategies. The application of machine learning interpretability techniques would be beneficial to demystify the decision-making process of the algorithms, aligning statistical findings with clinical insights. Additionally, research should focus on the development and validation of machine learning models in clinical settings to evaluate their practical utility in real-world preventive dentistry. Lastly, future studies should consider the socioeconomic and behavioral interventions suggested by the predictive models and assess their effectiveness in reducing the incidence of dental caries, thus moving from predictive analytics to actionable health outcomes.

## Data availability statement

The original contributions presented in the study are included in the article/supplementary material, further inquiries can be directed to the corresponding authors.

## Author contributions

S-AS-Z: Conceptualization, Data curation, Formal analysis, Funding acquisition, Investigation, Methodology, Project administration, Resources, Software, Supervision, Validation, Visualization, Writing – original draft, Writing – review & editing. MB: Data curation, Formal analysis, Investigation, Resources, Validation, Writing – original draft, Writing – review & editing. MS: Funding acquisition, Visualization, Writing – original draft, Writing – review & editing.
